# Epidermal Growth Factor Receptor (EGFR) mutation analysis, gene expression profiling and EGFR protein expression in primary prostate cancer

**DOI:** 10.1186/1471-2407-11-31

**Published:** 2011-01-25

**Authors:** Caterina Peraldo-Neia, Giorgia Migliardi, Maurizia Mello-Grand, Filippo Montemurro, Raffaella Segir, Ymera Pignochino, Giuliana Cavalloni, Bruno Torchio, Luciano Mosso, Giovanna Chiorino, Massimo Aglietta

**Affiliations:** 1 Department of Clinical Oncology, University of Torino Medical School, Institute for Cancer Research and Treatment, Candiolo, Turin, Italy; 2 Laboratory of Cancer Genomics, "Fondazione Edo ed Elvo Tempia Valenta", Biella, Italy; 3 Oncological Department, Medical Oncology, Institute for Cancer Research and Treatment (IRCC) Candiolo; 4 Histopathology Department, Mauriziano Hospital, Torino, Italy

## Abstract

**Background:**

Activating mutations of the epidermal growth factor receptor (*EGFR*) confer sensitivity to the tyrosine kinase inhibitors (TKi), gefitinib and erlotinib. We analysed EGFR expression, EGFR mutation status and gene expression profiles of prostate cancer (PC) to supply a rationale for EGFR targeted therapies in this disease.

**Methods:**

Mutational analysis of EGFR TK domain (exons from 18 to 21) and immunohistochemistry for EGFR were performed on tumour tissues derived from radical prostatectomy from 100 PC patients. Gene expression profiling using oligo-microarrays was also carried out in 51 of the PC samples.

**Results:**

EGFR protein overexpression (EGFR_high_) was found in 36% of the tumour samples, and mutations were found in 13% of samples. Patients with EGFR_high _tumours experienced a significantly increased risk of biochemical relapse (hazard ratio-HR 2.52, p=0.02) compared with patients with tumours expressing low levels of EGFR (EGFR_low_). Microarray analysis did not reveal any differences in gene expression between EGFR_high _and EGFR_low _tumours. Conversely, in EGFR_high _tumours, we were able to identify a 79 gene signature distinguishing mutated from non-mutated tumours. Additionally, 29 genes were found to be differentially expressed between mutated/EGFR_high _(n=3) and mutated/EGFR_low _tumours (n=5). Four of the down-regulated genes, U19/EAF2, ABCC4, KLK3 and ANXA3 and one of the up-regulated genes, FOXC1, are involved in PC progression.

**Conclusions:**

Based on our findings, we hypothesize that accurate definition of the EGFR status could improve prognostic stratification and we suggest a possible role for EGFR-directed therapies in PC patients. Having been generated in a relatively small sample of patients, our results warrant confirmation in larger series.

## Background

Prostate cancer (PC) is among the most frequently diagnosed solid tumours in men, and the metastatic forms still represent the second leading cause of cancer-related death [1,2]. Treatment of PC by radical prostatectomy, radiotherapy and anti-androgen therapy results in long term survival in patients with localized and androgen-dependent PC. By contrast, hormone-refractory prostate cancer (HRPC) forms are associated with disease relapse and poor patient survival [3,4]. At present, increasing serum prostate-specific antigen (PSA) levels following treatment of primary PC is used to identify PC biochemical relapse, a condition that anticipates clinically detectable tumour progression. The identification of novel biomarkers that predict the risk of relapse or that could be used as therapeutic targets is needed.

The molecular mechanisms responsible for PC development, progression and hormone-independence are not clear yet. Several findings suggest that alterations of different pathways involving growth factor receptors play a role in this multistep process [5,6]. In particular, the Epidermal Growth Factor Receptor (EGFR) is frequently overexpressed in PC and this is associated with a more aggressive clinical outcome. EGFR overexpression has also been linked to the transition from androgen-responsiveness to the androgen-independent/hormone-refractory phenotype [7,8]. Furthermore, preclinical data have suggested that the EGFR signalling pathway can activate the androgen receptor under conditions of clinical androgen deprivation [9]. EGFR has thus assumed considerable importance, due to overexpression in different tumour types and to its role as a drug target. A variety of anti-EGFR drugs are currently Food and Drug Administration-approved or under evaluation in clinical trials. These drugs include small inhibitory molecules such as gefitinib or erlotinib, as well as antibodies such as cetuximab and panitumumab. Gefitinib is an oral anilinoquinazolone compound that blocks the EGFR tyrosine kinase (TK) activity [10] resulting in the inhibition of downstream signalling pathways. Clinical evidence, mostly deriving from non small cell lung cancer (NSCLC) patients, demonstrated that activating mutations in the EGFR TK domain (exons from 18 to 21) predict response to gefitinib [11].

A recent study identified 4 novel missense mutations in exons 19, 20 and 21 of the EGFR TK domain in Korean and Caucasian PC patients. Three of them, G735S, G796S and E804G, led to an oncogenic activation promoting cell proliferation and invasion [12].

Preclinical studies have shown activity of gefitinib against PC cell lines and xenografts [13]. In a phase I clinical trial, 252 patients with different solid tumours, including 28 patients with HRPC, received oral gefitinib [14]. One patient with HRPC had a measurable reduction of disease in a lymph node metastasis, palliation of disease-related pain, and a reduction in PSA [15]. In another randomized phase II clinical trial 82 HRPC patients were treated with prednisone plus gefitinib or prednisone plus placebo [16]. This study showed limited antitumour activity of gefitinib in HRPC patients. However, patients were not selected on the basis of EGFR status. At present, no clinical data are available on EGFR-mutated PC patients treated with gefitinib.

In an effort to provide a rationale for further studies of targeted therapies in PC, we set out to analyze EGFR protein expression, EGFR mutations and their possible correlations with clinical parameters and outcomes. For 51 of these samples we also carried out gene expression profiling with oligo-microarrays.

## Methods

### Samples

One hundred glyofixx-fixed, paraffin-embedded PC specimens were retrieved after radical prostatectomy in 100 PC patients. According to the availability of fresh frozen tumour material, different subsets of the initial 100 patients selected for this analysis were studied. Immunohistochemistry and mutational analysis were performed in all the samples from 100 patients.

Fifty patients had tumour samples stored at -80°C and were submitted to gene profiling. For one of these patients, two different tumour samples were available. Therefore, the total number of samples submitted to gene profiling was 51.

The patients were of Italian origin and were diagnosed at ASL (Azienda Sanitaria Locale) 12, Hospital of Biella. To classify tumours according to grade, we used the definition proposed by Franiel et al [17]. Tumours with a Gleason Score from 4 to 6 were considered low grade and those with a Gleason Score from 7 to 9 were considered high grade.

### DNA extraction and direct sequencing

For all the specimens, it was possible to select tumour areas and extract DNA for EGFR mutational analysis. Genomic DNA was extracted from deparaffinised tumour tissue using the QIAamp DNA Mini Kit (Qiagen, Milan, Italy) following the manufacturer's instructions. The TK domain of EGFR coding sequence, from exon 18 to 21, was amplified by using primers and nested PCR conditions as previously described by Lynch et al [18]. PCR products were purified by QIAquick PCR purification kit (Qiagen) and sense and antisense sequences were obtained by using forward and reverse internal primers, respectively. Each exon was sequenced using the BigDye Terminator Cycle sequence following the PE Applied Biosystem strategy and Applied Biosystems ABI PRISM3100 DNA Sequencer (Applied Biosystem, Forster City, CA). All mutations were confirmed by performing two independent PCR amplifications.

### Immunohistochemistry

EGFR protein expression was evaluated on the entire cohort. Tissue sections (3 μm thick) were mounted on pre-coated slides, deparaffinised with xylene and rehydrated with graded ethanol. Endogenous peroxidase was blocked with Peroxidase Blocking Solution (DAKO, CA, USA) for 5 minutes. Sections were then incubated with primary antibody for EGFR (1:200, Clone: 31G7, Mouse anti-Human, Zymed) for one hour at room temperature. The reaction was visualised using EnVision staining kit (DAKO). Sections were counterstained with hemallume. About 100 cells were counted in three different fields. All slides were independently evaluated by two pathologists (L.M. and B.T.). Discordant cases were reviewed a third time and a consensus was reached. Basal EGFR expression (EGFR_low_) was defined as 10-49% of tumour cells staining positively for EGFR. High EGFR expression (EGFR_high_) was defined as ≥50% of tumour cells staining positively for EGFR.

### Microarray analysis

Gene expression profiling was evaluated for 51 samples from 50 patients (two specimens were analysed for one patient) as previously described [19]. Briefly, total RNA was isolated from sections of frozen tissues obtained from 50 radical prostatectomies collected between 2003-2005, using TriReagent (Sigma, St. Louis, MO, USA). A commercially available RNA sample from 32 normal prostates pooled from Caucasian males (ages: 21-50), constituted the prostate RNA Reference (Clontech, Mountain View, CA, USA). RNA quantity was evaluated by Bioanalyzer 2100 (Agilent Technologies, Palo Alto, CA, USA). Following the isolation procedure, mRNA was amplified starting from 5 μg of total RNA using MessageAmp aRNA Amplification kit (Ambion Inc. Austin TX, USA). Amino-allyl modified nucleotides were incorporated during the overnight *in vitro *transcription step according to the manufacturer's protocol. Labeling was performed using NHS (N-hydroxysuccinimidyl) ester Cy3 or Cy5 dyes (GE Healthcare Europe GMBH, Upsala-Sweden) able to react with the modified RNA. At least 5 μg of aaRNA for each sample were labeled and then purified with columns; 0.75 μg of labeled aaRNA for each sample were then hybridised. The Dye-Swap replication procedure was applied, in order to increase accuracy. Samples were hybridised on 22K human oligo-glass arrays, (Agilent Technologies). Arrays were scanned by Agilent scanner. Images obtained were analysed by the Feature Extraction software Agilent (version 9.5) and the text files were then processed using the Bioconductor package Limma (Linear models for microarray analysis). Class comparison and unsupervised hierarchical clustering were carried out using MeV (Multi Experiment Viewer) version 4.1 (http://www.tm4.org). In particular, non parametric testing (Wilcoxon, Rank Sum) was adopted for feature selection. Gene Ontology analysis was performed using the Database for Annotation, Visualization and Integrated Discovery (DAVID tool), (http://david.abcc.ncifcrf.gov).

### Real time quantitative PCR (qRT-PCR)

RNA was extracted and transcribed into cDNA using High capacity cDNA reverse transcription kit (PE Applied Biosystem). cDNA was then used for amplification of ABCC4, ANXA3, FOXC1, KLK3, U19 genes and PGK gene (housekeeping gene) with the following specific primers: FW ABCC4 5'-AGAGGGTGTCAGAGGCAATC-3', RV ABCC4 5'-CATCAAGTAGCAAAAAGGTCT-3'; FW ANXA3 5'- GTTGGACACCGAGGAACAGT, RV ANXA3 5'- GCTGTGCATTTGACCTCTCA-3'; FW FOXC1 5'- TAGCTGTCAAATGGCCTTC, RV FOXC1 5' - TAGTTCGGCTTTGAGGGTGT - 3'; FW KLK3 5'-TCCCAGACGTGGATTGGT-3', RV KLK3 5'-CAGGGTTGGGAATGGTTCT-3'; FW U19 5'- CAGGGAATTGTGTCTCAGGAC-3', RV U19 5'- GGCCACTGTTGTCTCGAAAT - 3'. Real-time PCR was carried out in triplicate in optical grade 96-well plates with 5 μl SYBR Green Master mix (PE Applied Biosystems), 12.5 μM of each primer, and 35 ng of cDNA in a volume of 60 μl. Thermal cycling was performed using the Applied Biosystems 7300 real-time PCR system with the following conditions: 95°C for 10 min, 40 cycles at 95°C for 15 sec, 60°C for 1 min. Relative quantitation of target genes was obtained by using the comparative cycle threshold (Ct) methods as previously described [20]. Briefly, to calculate the relative expression of the target gene mRNA normalized to PGK, the average of target C_t _was subtracted from the average of PGK C_t _(ΔC_t_). The amount of target, normalized to an endogenous reference and relative to a calibrator (fold-change) is given by 2^-ΔΔCt ^where the calculation of ΔΔCt involves subtraction by the ΔCt calibrator value (EGFR_low_). Assuming that ΔC_t _= 3.3 corresponds to a 10-fold difference of expression between PGK and the target, as calculated from log 10, ΔC_t _results were divided by 3.3 and represented on a logarithmic scale.

### Statistics

Comparisons between dichotomous variables were performed by the Fisher's exact test.

Time to biochemical relapse (TTBR) was calculated from the date of prostate cancer surgery to that of biochemical relapse, defined as a serum PSA level > 0.2 ng/ml in at least 2 subsequent measurements performed ≥3 months from prostatectomy [21]. Univariate comparisons of time to biochemical relapse (TTBR) according to EGFR immunohistochemical status, mutational status, disease stage and Gleason Score group were performed by Cox Proportional Hazards analysis and by drawing Kaplan-Meier curves, which were compared by the log-rank test. If more than one factor resulted significantly associated with TTBR at the p < 0.10 level, a multivariate Cox Proportional Hazards model including the significant factors was studied.

## Results

### Clinical parameters and EGFR expression on PC specimens

The characteristics of 100 patients with tumours available for analysis of EGFR expression and mutations are summarized in table1 and in additional file1, table S1.

**Table 1:** Patient characteristics

Variable	Value (%) n = 100
**Median age, years (range)**	66 (53-74)

**T status**	n = 98

T2	71 (72.44)

T3	26 (26.54)

T4	1 (1.02)

**Gleason Score**	n=100

G 4	1 (1)

G 5	5 (5)

G 6	12 (12)

G 7	52 (52)

G 8	22 (22)

G 9	8 (8)

**Figure 1:**
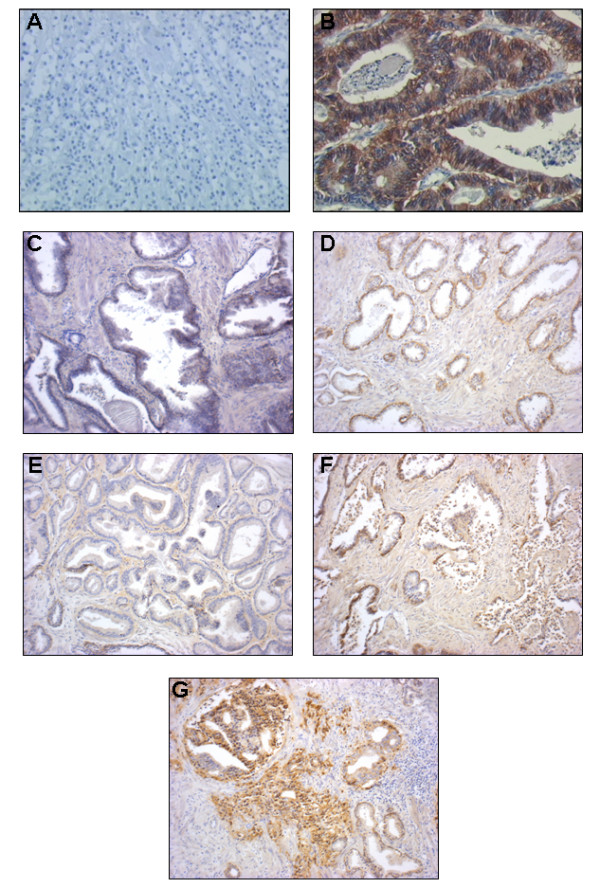
**Different EGFR pattern of expression in normal and PCs tissues revealed by immunohistochemistry**. **A**: negative control: lobular breast carcinoma not expressing EGFR. **B**: positive control: colon carcinoma expressing high levels of EGFR. **C**: normal prostate tissue not expressing EGFR. **D**: normal prostate tissue expressing EGFR. **E**: PC tissue not expressing EGFR. **F-G**: PC tissues expressing different pattern of EGFR. (Magnification 10X).

**Figure 2:**
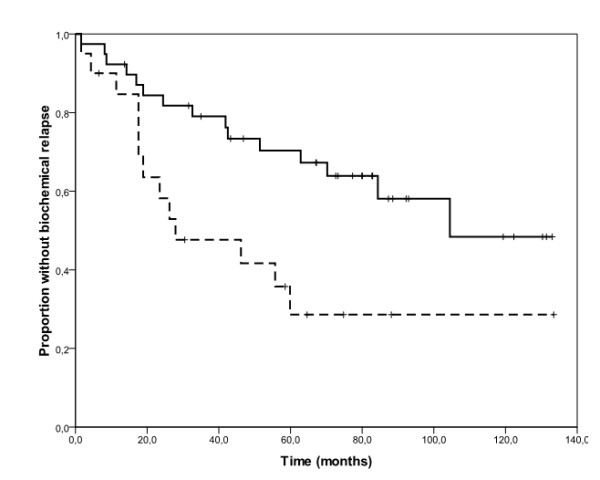
**Kaplan-Meier estimates of time to biochemical relapse according to EGFR immunohistochemical status**. The solid line represents patients with EGFR_low _tumours and the dashed line represents those with EGFR_high _tumours. Median time to biochemical relapse was 104 and 30 months for patients with EGFR_low_ and EGFR_high_ tumours, respectively (log-rank test, p=0.01).

**Figure 3:**
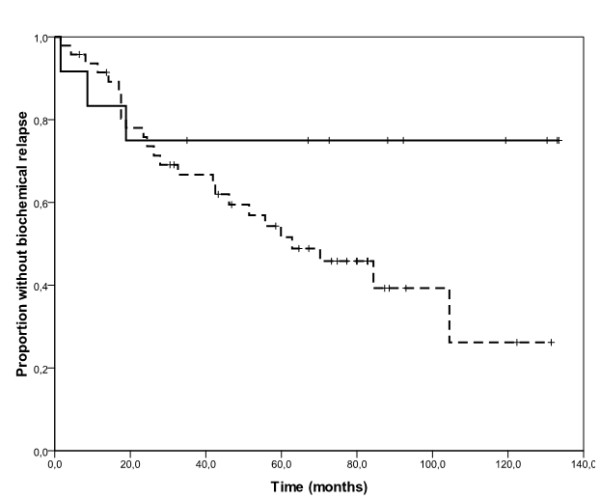
**Kaplan-Meier estimates of time to biochemical relapse according to Gleason Score category**. The solid line represents patients with low score tumours and the dashed line represents those with high score tumours. Median time to biochemical relapse was not reached for patients with low score tumours and was 63 months for those with high score tumours (log-rank test, p=0.08).

One hundred PC samples were evaluated for EGFR protein expression by immunohistochemistry. EGFR immunostaining was predominantly found in the membrane and the cytoplasm. EGFR was expressed at low levels in normal glands in 90 out of 100 samples (90%), and in 36 out of 100 cases (36%), tumour areas were classified as overexpressing EGFR (EGFR_high_) (additional file 1, table S1). Figure1 provides an example of the immunohistochemical pattern of EGFR protein expression in normal and tumour tissues. The impact of clinical and pathological variables on biochemical relapse was studied in a subgroup of 59 patients who underwent regular visits at the same Institution where they had undergone surgery, and for whom we had detailed follow-up information. The median follow-up for these patients was 207 months (range 16-366 months). The other 41 patients, who underwent follow-up visits at different Institutions after surgery, were excluded from this analysis because of incomplete or missing follow-up information. Twenty-eight out of 59 patients experienced biochemical relapse (overall median TTBR 84 months). Univariate analyses revealed a significant effect of EGFR immunohistochemical status on TTBR (figure2). Median TTBR was 104 and 30 months for patients with EGFR_low _and EGFR_high _tumours, respectively (hazard ratio-HR 2.53, p= 0.02). Furthermore, a strong trend towards a worse median TTBR was observed in patients with high vs low Gleason Score tumours (63 months vs not reached respectively, HR 2.80, p= 0.10) (figure3). Neither median age nor tumour stage at diagnosis correlated significantly with TTBR. When EGFR immunohistochemical status and Gleason Score were entered together in a Cox Proportional Hazards model, results did not change significantly, suggesting that EGFR immunohistochemical status (multivariate HR 2.67, p = 0.01) and Gleason Score (multivariate HR 3.04, p = 0.08) were independent predictors of TTBR.

### Mutational analysis

Of 100 patients who underwent surgical resection of their tumours, 13 presented point mutations (13%) in the TK domain of EGFR (Table 1). Specifically, we detected 2 point mutations in exon 19: T751I, previously described in erlotinib responsive NSCLC patients [22] and R748K, described in soft tissue sarcomas [23]. In exon 20, we found 5 point mutations: E804G, already described in PC [12], Q820R, G796V, P782L, F788L, not previously described. In exon 21, 4 novel missense mutations were revealed: L828M, F856Y, F856L, A839V, and 2 previously described, G863D, in partial gefitinib responsive NSCLC patients [24], and V851I, described in gefitinib-unresponsive NSCLC patients [25]. No point mutations were identified in exon 18. Table2 summarised all mutations found. Fifty-eight out of 100 specimens had the same silent mutation, both in heterozygotic and in homozygotic status, on the codon 787 in exon 20. No mutations were found in the normal counterpart. The frequency of mutations was not significantly different in EGFR_high _vs EGFR_low _tumours (6% vs 7%, respectively, p=0.50); all mutated samples had a high Gleason Score (13%), but no statistically significant association was revealed (p= 0.12). Finally, the mutation status had no significant impact on TTBR, although it should be noted that only 5 patients with mutated tumours were included in this analysis.

**Table 2:** Summary of EGFR mutations found in our work and respective references if previously described.

	EXON 19	
Patient 50	T751I	*Described by Tsao et al*

Patient 63 (P51)	R748K	*Described by Dobashi et al*

	**EXON 20**	

Patient 12	E804G	*Described by Cai et al*

Patient 64 (AA65)	Q820R	*Novel mutation*

Patient 72 (CC62)	P872L	*Novel mutation*

Patient 73 (CC75)	F788L	*Novel mutation*

Patient 99	G796V	*Novel mutation*

	**EXON 21**	

Patient 2	V851I	*Described by Cappuzzo et al*

Patient 8	G863D	*Described by Bell et al*

Patient 11	A839V	*Novel mutation*

Patient 40	L828M	*Novel mutation*

Patient 56	F856Y	*Novel mutation*

Patient 88	F856L	*Novel mutation*

### Gene profiling

Gene expression profile analysis was performed in 51 fresh frozen samples from 50 patients.

**Figure 4:**
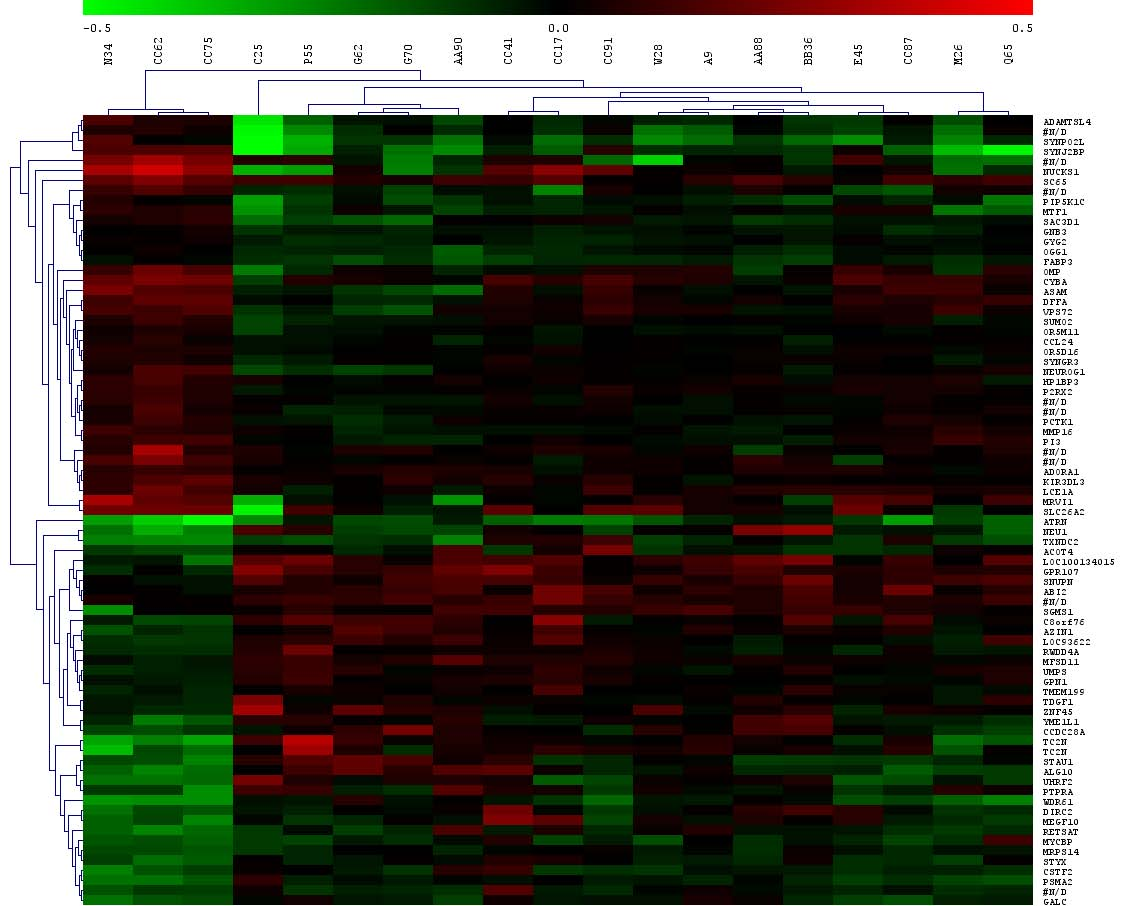
**Cluster analysis applied to EGFR**_**high**_**: a 79-gene signature separated EGFR**_**high**_**/mutated samples(N34, CC62, CC75)from EGFR**_**high**_**/WT samples**. (G70, C25, A9, E45, M26, AA88, AA90, BB36, CC17, CC41, CC87, CC91, G62, P55, Q65, W28). (N34, CC62, CC75, G70, C25, A9, E45, M26, AA88, AA90, BB36, CC17, CC41, CC87, CC91, G62, P55, Q65, W28 referred to patients code as in additional file 1, table S1)

Nineteen samples were EGFR_high _and 32 displayed a basal expression of EGFR. Comparison between these two classes did not give significant results in terms of differentially expressed genes. Analysing only the EGFR_high _samples, a 79-gene signature (40 up-regulated and 39 down-regulated) distinguished EGFR_high_/mutated samples from EGFR_high_/WT samples (figure 4 and additional file 2, table S2). In order to identify in which processes these genes are implicated, a Gene Ontology analysis was performed. Three biological processes are involved, namely cellular lipid metabolism, primary metabolism and the inflammatory response (Table 3).

**Table 3:** Gene Ontology of the 79 modulated probes in EGFR_high_/mutated compared to EGFR_high_/WT.

Category	Term	P-value	Genes (Entrez ID
GOTERM_BP_ALL	GO:0044255~cellular lipid metabolic process	0.03	122970, 2581, 54884, 23396, 2170, 259230

GOTERM_BP_ALL	GO:0044238~primary metabolic process	0.07	134, 122970, 5127, 1478, 4520, 2581, 7596, 54884, 115426, 10730, 4968, 4762, 7372, 6944, 26292, 23396, 84203, 8908, 6613, 2170, 4325, 259230, 51582, 6815, 63931, 6997, 10073, 5689, 1676, 5786

GOTERM_BP_ALL	GO:0006954~inflammatory response	0.09	134, 8455, 6369, 259230

**Figure 5:**
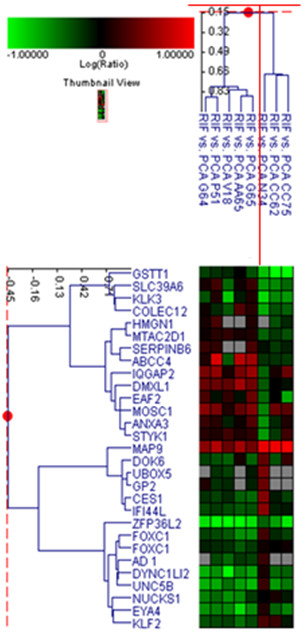
**Cluster analysis applied to 8 EGFR-mutated samples using the 29-gene signature distinguished two classes**. On the left branch, EGFR_low _samples and on the right branch EGFR_high _samples.

For 8 of the 13 cases harbouring an EGFR TK mutation fresh frozen tissue was available for gene expression analysis. In these samples we could identify a panel of 29 genes which were differentially expressed between EGFR_high _and EGFR_low _tumours (Table4). Figure5 shows the significant distinction of the two classes obtained from this 29-gene signature.

**Table 4:** Differentially expressed genes selected in samples mutated for EGFR TK domain.

EntrezGene	Gene Name	Description	**Log**_**10**_Ratio *
2952	**GSTT1**	Glutathione S-transferase theta-1	↓

10257	**ABCC4**	Multidrug resistance-associated protein 4	↓

1657	**DMXL1**	DmX-like protein 1	↓

25800	**SLC39A6**	Zinc transporter ZIP6 Precursor	↓

55840	**EAF2**	ELL-associated factor 2	↓

354	**KLK3**	Prostate-specific antigen Precursor (PSA)	↓

81035	**COLEC12**	Collectin-12 (Collectin placenta protein 1)	↓

10788	**IQGAP2**	Ras GTPase-activating-like protein	↓

123036	**MTAC2D1**	Tandem C2 domains nuclear protein	↓

3150	**HMGN1**	Non-histone chromosomal protein HMG-14	↓

55359	**STYK1**	Tyrosine protein-kinase	↓

64757	**MOSC1**	MOSC domain-containing protein 1, mitochondrial Precursor	↓

5269	**SERPINB6**	Serpin B6 (Placental thrombin inhibitor)	↓

1066	**ANXA3**	Annexin A3	↓

652708	**CES1**	Liver carboxylesterase 1 Precursor	↑

220164	**DOK6**	Docking protein 6 (Downstream of tyrosine kinase 6)	↑

2296	**FOXC1**	Forkhead box protein C1	↑

219699	**UNC5B**	etrin receptor UNC5B Precursor	↑

2070	**EYA4**	Eyes absent homolog 4	↑

10964	**IFI44L**	Interferon-induced protein 44-like	↑

678	**ZFP36L2**	Butyrate response factor 2	↑

64710	**NUCKS1**	Nuclear ubiquitous casein and cyclin-dependent kinases substrate (P1)	↑

10365	**KLF2**	Krueppel-like factor 2	↑

1783	**DYNC1LI2**	Cytoplasmic dynein 1 light intermediate chain 2	↑

79884	**MAP9**	Microtubule-associated protein 9	↑

2813	**GP2**	Pancreatic secretory granule membrane major glycoprotein GP2 Precursor	↑

22888	**UBOX5**	RING finger protein 37	↑

* Log_10_Ratio: logarithm (base 10) of the ratio between PC sample and prostate reference expression values. ↑: up regulated gene. ↓: down-regulated gene

**Figure 6:**
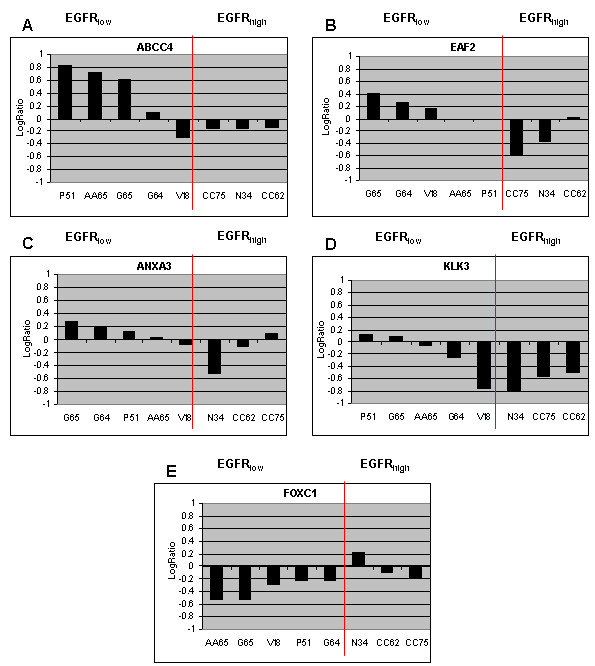
**Different modulation of selected genes between mutated and EGFR_low _(AA65, G64, G65, V18, P51) and mutated and EGFR_high _samples (N34, CC62, CC75) obtained by microarray analysis.** A significant distinction between EGFR_high _samples and EGFR_low _samples was found. (AA65, G64, G65, V18, P51, N34, CC62, CC75 referred to patients code as in additional file 1, table S1)

In particular, 5 of these genes are involved in prostate cancer progression and most of them are AR (androgen receptor) regulated. Among them, U19, ABCC4, ANXA3 and KLK3 are less expressed in EGFR_high _samples. One of the more expressed genes in EGFR_high _samples is FOXC1, a member of forkhead transcription factors (FOX) (figure6).

**Figure 7:**
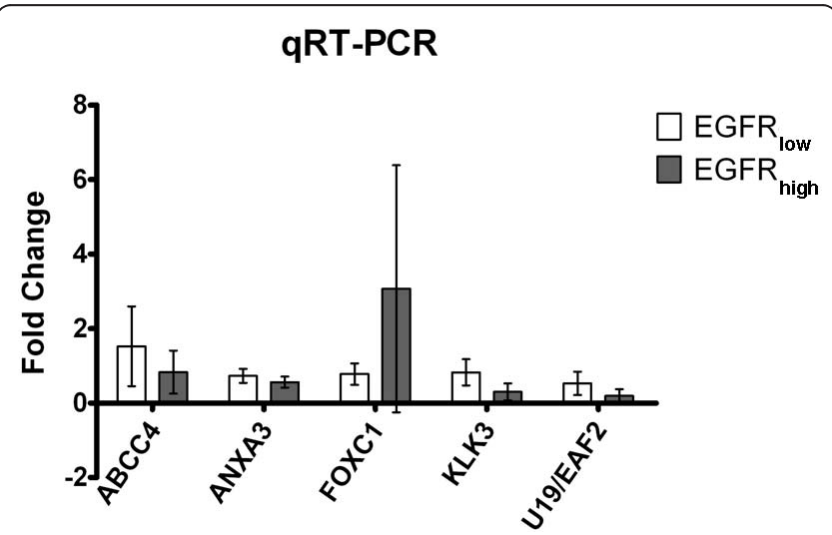
**Differentially expressed genes identified by microarray analysis were validated by relative qRT-PCR**. White bars represent the average mRNA levels of each gene in EGFR_low _class; gray bars represent the average mRNA levels of each gene in EGFR_high _class. Fold-change was calculated as described in "methods" section.

Quantitative RT-PCR on 8 EGFR mutated samples confirmed results obtained by microarray analysis on the five differentially expressed genes discussed above (figure 7).

## Discussion

In this study, we were interested in analysing EGFR expression and somatic mutations of its TK domain in prostatectomy specimens from patients with operable PC. Metastatic PC represents a challenge for the oncologist and novel therapeutic strategies are therefore warranted, especially when it evolves into the hormone-refractory status. Previous observations have pointed to deregulated EGFR function as a potentially relevant phenomenon in sustaining PC progression and the development of a hormone-refractory phenotype [7,8]. However, EGFR targeting agents have shown limited clinical activity in clinical trials conducted in PC patients [16,26-29]. Notably, these studies enrolled unselected patients with respect to EGFR status. EGFR targeting with TKIs, such as gefitinib and erlotinib [10,30], has proven successful in molecularly defined subsets of patients with non-small cell lung cancer (NSCLC), where specific mutations of the EGFR tyrosine kinase domain, but not protein expression, were predictive of the clinical efficacy [31]. Defining the relationship between EGFR status and the clinical behaviour of PC may therefore help define a possible therapeutic role of EGFR inhibitors in this patient subset.

In our study, we found that EGFR was expressed at normal levels in healthy prostate cells in 90% of the surgical specimens. In tumour areas, EGFR overexpression was found in 36 patient specimens (36%). In line with the literature, we confirmed that EGFR overexpression was significantly associated with biochemical relapse. Furthermore, although confirmation in a larger dataset is needed, EGFR overexpression predicts TTBR independently from Gleason Score, which is an established prognostic factor in this disease.

Having only analysed an Italian Caucasian population, and due to a small sample size, this incidence of EGFR overexpression is difficult to generalize. It has been shown, for example, that Afro-American patients affected by PC expressed EGFR at higher levels than Caucasian patients [32].

Mutational analysis revealed that 13 out of 100 patients had tumours carrying mutations in the EGFR TK domain. Two of these mutations (exon 19 T751I and exon 21 G863D) have been previously identified as predictors of response to small TKIs in NSCLC patients [22,23]. Another one, the V851I mutation, has been previously described in NSCLC patients refractory to TKIs treatment [24]. The E804G mutation that we found on exon 20, was previously described by Cai et al. in PC specimens [12]. These authors identified four novel somatic mutations in the EGFR tyrosine kinase domain of PC patients: G735S, G796S, E804G and R841K. They investigated their oncogenic potential by establishing stable clonal NIH3T3 cells expressing these mutations and WT EGFR to determine their ability to increase cell proliferation and invasion. Amongst them, the E804G mutation resulted as the most active and significant somatic missense mutation in the TK domain, being associated with the greatest growth potential, and by the highest transformation and invasion ability [12]. Q820R, G796V, P782L, F788L, A839V, L828M, F856Y, F856L are novel mutations and further exploration of their effect on the activation of the downstream EGFR pathway is warranted.

Gene expression profile analysis failed to identify genes that were differently expressed in tumours with high vs low levels of EGFR, or in EGFR mutated vsnon mutated tumours. The small sample size and the heterogeneity of mutations may have limited the ability of this analysis to capture significant gene expression patterns. Interestingly, when we restricted the analysis to EGFR-overexpressing tumours (EGFR_high_), we could identify a 79 gene signature distinguishing mutated from non mutated samples. Three biological processes (lipid and primary metabolic processes and the inflammatory response) are altered and they may help discriminate between the two classes. Further studies could reveal the role of EGFR mutations in the regulation of these specific genes. Another 29 genes were found to be differently modulated in mutated tumours according to their EGFR expression level (high vs low). Four of the genes that were expressed at lower levels in EGFR_high _samples have been previously linked to PC. The expression of U19/EAF2 in xenograft prostate tumours markedly induced apoptosis and inhibited tumour growth *in vivo *[33]. Moreover, the disruption of androgen-dependent growth regulation via U19/EAF2 down-regulation has been found to be commonly associated with PC progression. These observations suggest that U19/EAF2 may act as a tumour suppressor gene. Another finding revealed that the prostate specific ATP-binding cassette transporters ABCC4 was found to be expressed at higher levels in PC than in benign prostate tissue and decreased expression was found after androgen ablation [34]. Zhao et al. found a marked reduction in KLK3 expression levels in androgen-independent, compared with androgen-sensitive PC cell lines [35]. Finally, it was demonstrated that ANXA3 protein expression decreases from benign prostatic hypertrophy to localised pre-neoplastic lesions [36]. Among the genes that we found to be expressed at higher levels in EGFR_high _tumours, one has been previously linked to PC. FOXC1, a member of forkhead transcription factors (FOX), was found to be expressed at significantly high levels in androgen-independent PC xenografts [37]. Our data, which are mostly concordant with previous observations, suggest that EGFR overexpression may result in a more aggressive tumour behaviour [38], through deregulated function of these genes.

We acknowledge that these are preliminary data based on a small number of cases [12,39], with consequent limitations in the generalizability of our results. For example, the prevalence of EGFR mutations that we have found is in the range of that reported by other authors analyzing PC or cholangiocarcinoma [12,40]. Compared to other EGFR-driven diseases like NSCLC, where the prevalence of EGFR mutations has been reported to be as high as 30%, in PC EGFR mutations are less frequent. A larger sample size is therefore required to collect sufficient mutated cases to perform a more powerful analysis of the impact of mutations on prognosis or other biological features of tumours. Similarly, a larger number of mutated cases may result in the ability of gene expression analysis to capture significant gene expression patterns. In our study, because of the limited availability of fresh frozen material, the gene expression profile analysis was feasible in tumour samples from just 8 patients.

In summary, we found that 36% of the patients in this series had EGFR-overexpressing tumours and that this feature was significantly associated with biochemical relapse. Of the 13 tumours harbouring an EGFR mutation, all belonged to the high Gleason Score group. Of the identified mutations, some have been previously shown to predict the antitumor activity of small molecule tyrosine-kinase inhibitors. Further investigations on the novel mutations that we have identified may reveal new therapeutic targets. Finally, we identified a gene list in EGFR-mutated patients associated with EGFR expression.

## Conclusions

Although obtained in a small series of PC patients, our findings suggest that accurate definition of the EGFR status could improve prognostic stratification and suggests a possible role of EGFR-directed therapies in PC patients.

## Competing interests

All authors disclose any association that poses a conflict of interest in connection with the manuscript.

Microarray data are available on GEO (GSE 14206).

## Authors' contributions

CPN designed the study, carried out the experiments, and drafted the manuscript; GM performed PCR and mutational analysis; MMG performed microarray experiments; FM performed statistical analyses and reviewed the final version of the manuscript; RS performed immunohistochemistry experiments; YP and GC supervised the study and supported with data interpretation; BT and LM performed and analyzed the immunohistochemical data; GC performed statistical analysis of microarray data; MA participated in design and coordination of the study.

All authors have read and approved the final manuscript.

## Pre-publication history

The pre-publication history for this paper can be accessed here:

http://www.biomedcentral.com/1471-2407/11/31/prepub

## Supplementary Material

Additional file 1 **Table S1**. Clinical pathological characteristics and EGFR status in PC patients Click here for file

Additional file 2 **Table S2**. 79-gene signature (40 up-regulated and 39 down-regulated) distinguishing EGFR_high_/mutated samples from EGFR_high_/WT samples Click here for file

## References

[B1] Gronberg H Prostate cancer epidemiology Lancet 2003 361 859 864 10.1016/S0140-6736(03)12713-4 12642065

[B2] Jemal A Tiwari RC Murray T Ghafoor A Samuels A Ward E American Cancer Society Cancer statistics. CA Cancer J Clin 2004 54 8 29 10.3322/canjclin.54.1.8 14974761

[B3] Albertsen PC Hanley JA Fine J 20-year outcomes following conservative management of clinically localized prostate cancer JAMA 2005 293 2095 2101 10.1001/jama.293.17.2095 15870412

[B4] Roth BJ Prostate cancer chemotherapy: emerging from the shadows J Clin Oncol 2005 23 3302 3303 10.1200/JCO.2005.11.933 15738529

[B5] Mimeault M Batra SK Recent advances on multiple tumorigenic cascades involved in prostatic cancer progression and targeting therapies Carcinogenesis 2006 27 1 22 10.1093/carcin/bgi229 16195239

[B6] Djakiew D Dysregulated expression of growth factors and their receptors in the development of prostate cancer Prostate 2000 42 150 160 10.1002/(SICI)1097-0045(20000201)42:2<150::AID-PROS10>3.0.CO;2-H 10617873

[B7] Shah RB Ghosh D Elder JT Epidermal growth factor receptor (ErbB1) expression in prostate cancer progression: correlation with androgen independence Prostate 2006 66 1437 44 10.1002/pros.20460 16741920

[B8] Di Lorenzo G Tortora G D'Armiento FP De Rosa G Staibano S Autorino R D'Armiento M De Laurentiis M De Placido S Catalano G Bianco AR Ciardiello F Expression of epidermal growth factor receptor correlates with disease relapse and progression to androgen-independence in human prostate cancer Clinical Cancer Res 2002 8 3438 3444 12429632

[B9] Pignon JC Koopmansch B Nolens G Delacroix L Waltregny D Winkler R Androgen receptor controls EGFR and ERBB2 gene expression at different levels in prostate cancer cell lines Cancer Res 2009 69 2941 2949 10.1158/0008-5472.CAN-08-3760 19318561

[B10] Ciardiello F De Vita F Orditura M De Placido S Tortora G Epidermal growth factor receptor tyrosine kinase inhibitors in late stage clinical trials Expert Opin Emerg Drugs 2003 8 501 514 10.1517/14728214.8.2.501 14662002

[B11] Dahabreh IJ Linardou H Siannis F Kosmidis P Bafaloukos D Murray S Somatic EGFR mutation and gene copy gain as predictive biomarkers for response to tyrosine kinase inhibitors in non-small cell lung cancer Clin Cancer Res 2010 16 291 303 10.1158/1078-0432.CCR-09-1660 20028749

[B12] Cai CQ Peng Y Buckley MT Wei J Chen F Liebes L Gerald WL Pincus MR Osman I Lee P Epidermal growth factor receptor activation in prostate cancer by three novel missense mutations Oncogene 2008 27 3201 3210 10.1038/sj.onc.1210983 18193092

[B13] Taguchi F Koh Y Koizumi F Tamura T Saijo N Nishio K Anticancer effects of ZD6474, a VEGF receptor tyrosine kinase inhibitor, in gefitinib ("Iressa")-sensitive and resistant xenograft models Cancer Sci 2004 95 984 989 10.1111/j.1349-7006.2004.tb03187.x 15596048 PMC11159739

[B14] Lorusso PM Phase I studies of ZD1839 in patients with common solid tumors Semin Oncol 2003 30 1 Suppl 1 21 29 10.1053/sonc.2003.50029 12644981

[B15] Wilding G Soulie P Trump D Das-Gupta A Small E Results from a pilot Phase I trial of gefitinib combined with docetaxel and estramustine in patients with hormone-refractory prostate cancer Cancer 2006 106 1917 1924 10.1002/cncr.21831 16568471

[B16] Boccardo F Rubagotti A Conti G Battaglia M Cruciani G Manganelli A Ricc S Lapini A Prednisone plus gefitinib versus prednisone plus placebo in the treatment of hormone-refractory prostate cancer: a randomized phase II trial Oncology 2008 74 223 228 10.1159/000151391 18714171

[B17] Franiel T Lüdemann L Taupitz M Rost J Asbach P Beyersdorff D Pharmacokinetic MRI of the prostate: parameters for differentiating low-grade and high-grade prostate cancer Rofo 2009 181 536 42 19353483 10.1055/s-0028-1109168

[B18] Lynch TJ Bell DW Sordella R Gurubhagavatula S Okimoto RA Brannigan BW Harris PL Haserlat SM Supko JG Haluska FG Louis DN Christiani DC Settleman J Haber DA Activating mutations in the epidermal growth factor receptor underlying responsiveness of non-small-cell lung cancer to gefitinib N Engl J Med 2004 350 2129 2139 10.1056/NEJMoa040938 15118073

[B19] Cangemi R Mensah A Albertini V Jain A Mello-Grand M Chiorino G Catapano CV Carbone GM Reduced expression and tumor suppressor function of the ETS transcription factor ESE-3 in prostate cancer Oncogene 2008 27 2877 2885 10.1038/sj.onc.1210953 18037958

[B20] Cavalloni G Danè A Piacibello W Bruno S Lamas E Bréchot C Aglietta M The involvement of human-nuc gene in polyploidization of K562 cell line Exp Hematol 2000 28 1432 1440 10.1016/S0301-472X(00)00558-0 11146165

[B21] Cronin AM Godoy G Vickers AJ Definition of biochemical recurrence after radical prostatectomy does not substantially impact estimates for prognostic factors J Urol 2010 183 984 989 10.1016/j.juro.2009.11.027 20083281 PMC2919806

[B22] Tsao MS Sakurada A Cutz JC Zhu CQ Kamel-Reid S Squire J Lorimer I Zhang T Liu N Daneshmand M Marrano P da Cunha Santos G Lagarde A Richardson F Seymour L Whitehead M Ding K Pater J Shepherd FA Erlotinib in lung cancer - molecular and clinical predictors of outcome N Engl J Med 2005 14 133 144 Erratum in: *N Engl J Med. *2006;**16**:1746 10.1056/NEJMoa050736 16014883

[B23] Dobashi Y Suzuki S Sugawara H Ooi A Involvement of epidermal growth factor receptor and downstream molecules in bone and soft tissue tumors Hum Pathol 2007 38 914 925 10.1016/j.humpath.2006.12.005 17376509

[B24] Bell DW Lynch TJ Haserlat SM Harris PL Okimoto RA Brannigan BW Sgroi DC Muir B Riemenschneider MJ Iacona RB Krebs AD Johnson DH Giaccone G Herbst RS Manegold C Fukuoka M Kris MG Baselga J Ochs JS Haber DA Epidermal growth factor receptor mutations and gene amplification in non-small-cell lung cancer: molecular analysis of the IDEAL/INTACT gefitinib trials J Clin Oncol 2005 23 31 8081 8092 Epub 2005 Oct 3 10.1200/JCO.2005.02.7078 16204011

[B25] Cappuzzo F Hirsch FR Rossi E Bartolini S Ceresoli GL Bemis L Haney J Witta S Danenberg K Domenichini I Ludovini V Magrini E Gregorc V Doglioni C Sidoni A Tonato M Franklin WA Crino L Bunn PA Jr Varella-Garcia M **Epidermal growth factor receptor gene and protein and gefitinib sensitivity in non-small-cell lung cancer**. *J Natl Cancer Inst* 2005 97 9 643 655 15870435 10.1093/jnci/dji112

[B26] Pezaro C Rosenthal MA Gurney H Davis ID Underhill C Boyer MJ Kotasek D Solomon B Toner GC An open-label, single-arm phase two trial of gefitinib in patients with advanced or metastatic castration-resistant prostate cancer Am J Clin Oncol 2009 32 338 41 10.1097/COC.0b013e31818b946b 19363437

[B27] Small EJ Fontana J Tannir N DiPaola RS Wilding G Rubin M Iacona RB Kabbinavar FF A phase II trial of gefitinib in patients with non-metastatic hormone-refractory prostate cancer BJU Int 2007 100 765 9 10.1111/j.1464-410X.2007.07121.x 17822457

[B28] Sridhar SS Hotte SJ Chin JL Hudes GR Gregg R Trachtenberg J Wang L Tran-Thanh D Pham NA Tsao MS Hedley D Dancey JE Moore MJ A Multicenter Phase II Clinical Trial of Lapatinib (GW572016) in Hormonally Untreated Advanced Prostate Cancer Am J Clin Oncol 2009 in press 10.1097/COC.0b013e3181beac33 20042973

[B29] de Bono JS Bellmunt J Attard G Droz JP Miller K Flechon A Sternberg C Parker C Zugmaier G Hersberger-Gimenez V Cockey L Mason M Graham J Open-label phase II study evaluating the efficacy and safety of two doses of pertuzumab in castrate chemotherapy-naive patients with hormone-refractory prostate cancer J Clin Oncol 2007 25 257 62 10.1200/JCO.2006.07.0888 17235043

[B30] Prewett M Rockwell P Rockwell RF Giorgio NA Mendelsohn J Scher HI Goldstein NI The biologic effects of C225, a chimeric monoclonal antibody to the EGFR, on human prostate carcinoma J Immunother Emphasis Tumor Immunol 1996 19 419 427 9041461 10.1097/00002371-199611000-00006

[B31] Parra HS Cavina R Latteri F Zucali PA Campagnoli E Morenghi E Grimaldi GC Roncalli M Santoro A Analysis of epidermal growth factor receptor expression as a predictive factor for response to gefitinib ('Iressa', ZD1839) in non-small-cell lung cancer Br J Cancer 2004 91 208 212 15187994 10.1038/sj.bjc.6601923 PMC2409824

[B32] Shuch B Mikhail M Satagopan J Lee P Yee H Chang C Cordon-Cardo C Taneja SS Osman I Racial disparity of epidermal growth factor receptor expression in prostate cancer J Clin Oncol 2004 22 4725 4729 10.1200/JCO.2004.06.134 15570072

[B33] Xiao W Zhang Q Jiang F Pins F Kozlowski JM Wang Z Suppression of prostate tumor growth by U19, a novel testosterone-regulated apoptosis inducer Cancer Res 2003 63 4698 4704 12907652

[B34] Ho LL Kench JG Handelsman DJ Scheffer GL Stricker PD Grygiel JG Sutherland RL Henshall SM Allen JD Horvath LG Androgen regulation of multidrug resistance-associated protein 4 (MRP4/ABCC4) in prostate cancer Prostate 2008 68 1421 1429 10.1002/pros.20809 18615486

[B35] Zhao H Kim Y Wang P Lapointe J Tibshirani R Pollack JR Brooks JD Genome-wide characterization of gene expression variations and DNA copy number changes in prostate cancer cell lines Prostate 2005 63 187 197 10.1002/pros.20158 15486987

[B36] Köllermann J Schlomm T Bang H Schwall GP von Eichel-Streiber C Simon R Schostak M Huland H Berg W Sauter G Klocker H Schrattenholz A Expression and prognostic relevance of annexin A3 in prostate cancer Eur Urol 2008 54 1314 1323 18222597 10.1016/j.eururo.2008.01.001

[B37] van der Heul-Nieuwenhuijsen L Dits NF Jenster G Gene expression of forkhead transcription factors in the normal and diseased human prostate BJU Int 2009 103 1574 1580 10.1111/j.1464-410X.2009.08351.x 19220249

[B38] de Muga S Hernández S Agell L Salido M Juanpere N Lorenzo M Lorente JA Serrano S Lloreta J Molecular alterations of EGFR and PTEN in prostate cancer: association with high-grade and advanced-stage carcinomas Mod Pathol 2010 23 703 12 10.1038/modpathol.2010.45 20208477

[B39] Douglas DA Zhong H Ro JY Oddoux C Berger AD Pincus MR Satagopan JM Gerald WL Scher HI Lee P Osman I Novel mutations of epidermal growth factor receptor in localized prostate cancer Front Biosci 2006 11 2518 25 10.2741/1986 16720329

[B40] Leone F Cavalloni G Pignochino Y Sarotto I Ferraris R Piacibello W Venesio T Capussotti L Risio M Aglietta M Somatic mutations of epidermal growth factor receptor in bile duct and gallbladder carcinoma Clin Cancer Res 2006 12 1680 1685 10.1158/1078-0432.CCR-05-1692 16551849

