# Supplementary material for: Epidermal Growth Factor Receptor (EGFR) mutation analysis, gene expression profiling and EGFR protein expression in primary prostate cancer
Source: BMC Cancer. 2011 Jan 25;11:31. doi: 10.1186/1471-2407-11-31 (PMC3040720; doi:10.1186/1471-2407-11-31)
Supplement: Additional file 1 — Table S1. Clinical pathological characteristics and EGFR status in PC patients [file 1471-2407-11-31-S1.DOC]

| **Patient (microarray code)** | **Age** | **TNM** | **Gleason Score** | **Relapse status *** | **EGFR over-expression **** | **EGFR Mutations** |
| --- | --- | --- | --- | --- | --- | --- |
| **1** | **68** | **T2a N0 M0** | **7** | **0** | **low** | **-** |
| **2** | **67** | **T2c N0 M0** | **7** | **1** | **low** | **Ex 21 V851I** |
| **3** | **74** | **T2c N0 M0** | **8** | **NA** | **low** | **-** |
| **4** | **66** | **T2c N0 M0** | **8** | **1** | **high** | **-** |
| **5** | **60** | **T2c N0 M0** | **8** | **1** | **high** | **-** |
| **6** | **64** | **T2a N0 M0** | **5** | **0** | **low** | **-** |
| **7** | **73** | **T2c N0 M0** | **8** | **1** | **low** | **-** |
| **8** | **66** | **T2c N0 M0** | **8** | **1** | **high** | **Ex 21 G863D** |
| **9** | **67** | **T2c N0 M0** | **8** | **1** | **low** | **-** |
| **10** | **63** | **T3a N0 M0** | **6** | **0** | **high** | **-** |
| **11** | **67** | **T2a N0 M0** | **8** | **0** | **low** | **Ex 21 A839V** |
| **12** | **68** | **T3a N0 Mx** | **7** | **persistence** | **high** | **Ex 20 E804G** |
| **13** | **75** | **T2c N0 M0** | **9** | **0** | **high** | **-** |
| **14** | **60** | **T2c N0 M0** | **8** | **1** | **high** | **-** |
| **15** | **66** | **T2c N0 M0** | **8** | **1** | **high** | **-** |
| **16** | **59** | **T2c N0 M0** | **7** | **1** | **low** | **-** |
| **17** | **67** | **T2a N0 M0** | **4** | **1** | **low** | **-** |
| **18** | **54** | **T2c N0 M0** | **7** | **persistence** | **low** | **-** |
| **19** | **64** | **T2a N0 M0** | **6** | **1** | **high** | **-** |
| **20** | **68** | **T2a N0 M0** | **5** | **NA** | **high** | **-** |
| **21** | **71** | **T2a N0 M0** | **8** | **1** | **low** | **-** |
| **22** | **58** | **T2c N0 M0** | **7** | **1** | **low** | **-** |
| **23** | **65** | **T2a N0 M0** | **7** | **1** | **high** | **-** |
| **24** | **70** | **T2a N0 M0** | **5** | **0** | **low** | **-** |
| **25** | **64** | **T2b N0 M0** | **5** | **0** | **low** | **-** |
| **26** | **65** | **T2c N0 M0** | **8** | **0** | **low** | **-** |
| **27** | **67** | **T3a N0 M0** | **7** | **0** | **low** | **-** |
| **28** | **73** | **T2c N0 M0** | **7** | **0** | **low** | **-** |
| **29** | **69** | **T2b N0 M0** | **8** | **NA** | **low** | **-** |
| **30** | **67** | **T2b N0 M0** | **6** | **0** | **low** | **-** |
| **31** | **68** | **T2b N0 M0-x** | **8** | **1** | **high** | **-** |
| **32** | **71** | **T2a Nx M0** | **7** | **0** | **low** | **-** |
| **33** | **59** | **T2b N0 M0** | **8** | **0** | **low** | **-** |
| **34** | **66** | **T2c N0 M0** | **8** | **0** | **low** | **-** |
| **35** | **69** | **T2b Nx M0** | **7** | **1** | **low** | **-** |
| **36 (D40)** | **50** | **T3a N0 M0** | **7** | **0** | **low** | **-** |
| **37 (A1)** | **62** | **T2c N0 M0** | **7** | **0** | **low** | **-** |
| **38 (G70)** | **73** | **T2b Nx M0** | **7** | **1** | **high** | **-** |
| **39 (BB5)** | **61** | **T2c N0 M0** | **7** | **1** | **low** | **-** |
| **40 (V18)** | **68** | **T2c Nx M0** | **7** | **0** | **low** | **Ex 21 L828M** |
| **41 (C25)** | **65** | **T3a N0 M0** | **7** | **1** | **high** | **-** |
| **42 (E50)** | **67** | **T3a N0 M0** | **8** | **NA** | **low** | **-** |
| **43 (I81)** | **62** | **T3a N0 M0** | **7** | **NA** | **low** | **-** |
| **44** | **52** | **T2c Nx M0** | **6** | **NA** | **low** | **-** |
| **45 (B11)** | **68** | **T2c Nx M0** | **7** | **NA** | **low** | **-** |
| **46 (D36)** | **73** | **T3a Nx M0** | **7** | **0** | **low** | **-** |
| **47 (B12)** | **72** | **T2c Nx M0** | **6** | **persistence** | **low** | **-** |
| **48 (P57)** | **69** | **T2c Nx M0** | **8** | **1** | **low** | **-** |
| **49 (A9)** | **67** | **T2b N0 M0** | **6** | **1** | **high** | **-** |
| **50** | **64** | **T2c N0 M0** | **8** | **NA** | **low** | **Ex 19 T751I** |
| **51 (P60)** | **53** | **T2c Nx M0** | **5** | **persistence** | **low** | **-** |
| **52** | **57** | **T2c Nx M0** | **6** | **NA** | **high** | **-** |
| **53 (E45)** | **60** | **T2c N0 M0** | **6** | **0** | **high** | **-** |
| **54 (A5)** | **70** | **T3a N0 M0** | **8** | **Persistence** | **low** | **-** |
| **55 (M26)** | **61** | **T2c Nx M0** | **7** | **0** | **high** | **-** |
| **56 (N34)** | **69** | **T3b Nx M0** | **7** | **Persistence** | **high** | **Ex 21 F856Y** |
| **57 (F55)** | **60** | **T2c Nx M0** | **7** | **0** | **low** | **-** |
| **58 (B17)** | **71** | **T2b Nx M0** | **7** | **1** | **low** | **-** |
| **59 (I88)** | **63** | **T3b N0 M0** | **9** | **NA** | **low** | **-** |
| **60 (A3)** | **67** | **T2b N0 M0** | **7** | **1** | **low** | **-** |
| **61 (P53)** | **72** | **T3a N0 M0** | **7** | **0** | **low** | **-** |
| **62 (W25)** | **69** | **T3a N0 M0** | **8** | **NA** | **low** | **-** |
| **63 (P51)** | **53** | **T4 N1 M0** | **7** | **persistence** | **low** | **Ex 19 R748K** |
| **64 (AA65)** | **69** | **T2c N0 M0** | **7** | **NA** | **low** | **Ex 20 Q820R** |
| **65 (AA88)** | **69** | **T2c Nx M0** | **7** | **0** | **high** | **-** |
| **66 (AA90)** | **65** | **T2c N0 M0** | **7** | **1** | **high** | **-** |
| **67 (BB36)** | **64** | **T2b N0 M0** | **7** | **1** | **high** | **-** |
| **68 (CC3)** | **55** | **T3b N0 M0** | **7** | **NA** | **low** | **-** |
| **69 (CC17)** | **63** | **T3a N0 M0** | **8** | **persistence** | **high** | **-** |
| **70 (CC35)** | **65** | **T2c Nx M0** | **6** | **0** | **low** | **-** |
| **71 (CC41)** | **67** | **T2c Nx Mx** | **7** | **NA** | **high** | **-** |
| **72 (CC62)** | **70** | **T2c N0 M0** | **7** | **0** | **high** | **Ex 20 P782L** |
| **73 (CC75)** | **66** | **T3b Nx Mx** | **7** | **NA** | **high** | **Ex 20 F788L** |
| **74 (CC83)** | **67** | **T2c Nx Mx** | **7** | **0** | **low** | **-** |
| **75 (CC87)** | **70** | **T3b N0 Mx** | **9** | **persistence** | **high** | **-** |
| **76 (CC91)** | **71** | **T3b pN0 M0** | **7** | **0** | **high** | **-** |
| **77** | **64** | **T3a Nx Mx** | **7** | **NA** | **high** | **-** |
| **78** | **77** | **T3b Nx Mx** | **9** | **NA** | **high** | **-** |
| **79** | **72** | **T2c Nx Mx** | **7** | **NA** | **low** | **-** |
| **80** | **59** | **NA** | **6** | **NA** | **low** | **-** |
| **81** | **73** | **T3a N0 Mx** | **7** | **NA** | **low** | **-** |
| **82** | **71** | **T3b N0 Mx** | **7** | **NA** | **high** | **-** |
| **83** | **69** | **NA** | **8** | **NA** | **low** | **-** |
| **84** | **76** | **T3a NxMx** | **7** | **NA** | **low** | **-** |
| **85 (G62)** | **74** | **T2c N0 M0** | **9** | **NA** | **high** | **-** |
| **86 (G64)** | **70** | **T3a N0 M0** | **9** | **NA** | **low** | **-** |
| **87 (H75)** | **70** | **T2b Nx M0** | **7** | **0** | **low** | **-** |
| **88 (K6)** | **67** | **T2c N0 M0** | **9** | **0** | **low** | **Ex 21 F856L** |
| **89 (L12)** | **65** | **T2c N0 M0** | **7** | **1** | **low** | **-** |
| **90 (L16)** | **72** | **T2c N0 M0** | **7** | **0** | **low** | **-** |
| **91 (M21)** | **62** | **T2 Nx M0** | **7** | **1** | **low** | **-** |
| **92** | **66** | **T2c N1 M0** | **9** | **NA** | **high** | **-** |
| **93 (P55)** | **72** | **T2c Nx M0** | **7** | **NA** | **high** | **-** |
| **94 (Q65)** | **72** | **T2c N0 M0** | **7** | **NA** | **high** | **-** |
| **95 (CRY0)** | **68** | **T2c Nx M0** | **7** | **1** | **low** | **-** |
| **96 (W28)** | **61** | **T2c Nx M0** | **6** | **NA** | **high** | **-** |
| **97 (X40)** | **68** | **T2a Nx M0** | **6** | **0** | **low** | **-** |
| **98** | **63** | **T2a Nx M0** | **7** | **NA** | **low** | **-** |
| **99** | **64** | **T3a Nx M0** | **7** | **NA** | **low** | **Ex 20 G796V** |
| **100** | **65** | **T3b Nx M0** | **7** | **NA** | **low** | **-** |

*Relapse status: 0: non relapser 1: relapser. NA: not available. Persistence: disease persistence.

**EGFR expression. High: High expression +;Low: basal expression -
